# Association between Functional Variables and Heart Failure after Myocardial Infarction in Rats

**DOI:** 10.5935/abc.20160015

**Published:** 2016-02

**Authors:** Bertha F. Polegato, Marcos F. Minicucci, Paula S. Azevedo, Andréa F. Gonçalves, Aline F. Lima, Paula F. Martinez, Marina P. Okoshi, Katashi Okoshi, Sergio A. R. Paiva, Leonardo A. M. Zornoff

**Affiliations:** Faculdade de Medicina de Botucatu - Universidade Estadual Paulista "Júlio de mesquita Filho" - UNESP Botucatu, SP - Brazil

**Keywords:** Heart Failure / complications, Myocardial Infarction, Rats, Ventricular Dysfunction

## Abstract

**Background:**

Heart failure prediction after acute myocardial infarction may have important clinical implications.

**Objective:**

To analyze the functional echocardiographic variables associated with heart failure in an infarction model in rats.

**Methods:**

The animals were divided into two groups: control and infarction. Subsequently, the infarcted animals were divided into groups: with and without heart failure. The predictive values were assessed by logistic regression. The cutoff values predictive of heart failure were determined using ROC curves.

**Results:**

Six months after surgery, 88 infarcted animals and 43 control animals were included in the study. Myocardial infarction increased left cavity diameters and the mass and wall thickness of the left ventricle. Additionally, myocardial infarction resulted in systolic and diastolic dysfunction, characterized by lower area variation fraction values, posterior wall shortening velocity, E-wave deceleration time, associated with higher values of E / A ratio and isovolumic relaxation time adjusted by heart rate. Among the infarcted animals, 54 (61%) developed heart failure. Rats with heart failure have higher left cavity mass index and diameter, associated with worsening of functional variables. The area variation fraction, the E/A ratio, E-wave deceleration time and isovolumic relaxation time adjusted by heart rate were functional variables predictors of heart failure. The cutoff values of functional variables associated with heart failure were: area variation fraction < 31.18%; E / A > 3.077; E-wave deceleration time < 42.11 and isovolumic relaxation time adjusted by heart rate < 69.08.

**Conclusion:**

In rats followed for 6 months after myocardial infarction, the area variation fraction, E/A ratio, E-wave deceleration time and isovolumic relaxation time adjusted by heart rate are predictors of heart failure onset.

## Introduction

Heart failure syndrome is considered a public health problem with important prognostic implications. In this sense, around 50% of patients with cardiac dysfunction die within 5 years. In addition, 40% of patients die during the period of 1 year after the first hospitalization for heart failure, with many of the deaths occurring as sudden death.^1,2^

It is currently believed that the myocardial infarction (MI) is the main etiology of ventricular dysfunction. In this sense, epidemiological studies suggest that the signs and symptoms of heart failure are present in 25% of MI cases. Moreover, approximately 40% of MI cases are accompanied by systolic alterations in the left ventricle (LV). Recently, it was verified that 10% of MI patients have a restrictive pattern, suggesting severe diastolic dysfunction.^3^ Thus, the association between MI and ventricular dysfunction cannot be neglected.

One of the most often used strategies for the study of functional alterations caused by coronary occlusion is the use of the experimental infarction model in rats. Among other factors, this is due to the low cost and simplicity of handling these animals. The most important factor, however, refers to the similarity with the physiopathological changes that occur after an infarction in humans.^4^

Echocardiography has been widely used in the study of the morphological and functional alterations after coronary occlusion.^5-18^ However, there has been no consensus on which functional variables are predictive of heart failure in this model. Thus, our objective was to evaluate the functional variables associated with heart failure in the model. In addition, we intend to determine the critical values for heart failure prediction for each variable.

## Methods

The experimental protocol of this study was approved by the Animal Experimentation Ethics Committee of our institution, complying with the Ethical Principies in Animal Experimentation adopted by the Brazilian College of Animal Experimentation.

### Experimental infarction

Male Wistar rats, weighing between 200 and 250 g were studied. Acute myocardial infarction was produced according to the previously described method.^19,20^ Briefly, the rats were anesthetized with ketamine (70 mg/kg) and xylazine (5 mg/kg) and submitted to a left lateral thoracotomy. After exteriorization of the heart, the left atrium was moved away and the left coronary artery was ligated with a 5-0 monofilament nylon suture between the pulmonary artery outflow tract and the left atrium. Subsequently, the heart was returned to the chest, the lungs were inflated with positive pressure and the thorax closed using cotton 10 sutures. Coronary occlusion was not performed in 43 animals (Control Group).

The animals were kept in cages for recovery, fed standard commercial chow and had free access to water, with regular 12 hour light: dark cycles, at a temperature of approximately 25° C and controlled humidity.

### Echocardiographic study

Echocardiography was performed 6 months after the infarction. The animals were anesthetized intramuscularly with ketamine (50 mg/kg) and xylazine (1 mg/kg), for the echocardiographic study. After trichotomy of the anterior chest region, the animals were positioned in the supine position in a specially designed grooves that allows slight left lateral rotation for the examination, using Philips equipment (HDI 5000 model) equipped with a multi-frequency electronic transducer up to 12 MH. All measurements were made in accordance with the recommendations of the American Society of Echocardiography/European Association of Echocardiography.^21^ Left ventricular cavity image was obtained by positioning the M-mode cursor between the papillary muscles, right below the mitral valve plane. The LV diastolic diameter (LVDD) and left ventricular septal thickness (LVST) were measured at the moment corresponding to the maximum cavity diameter. The LV Systolic diameter (LVSD) was measured at the maximum systolic excursion of the posterior wall of the cavity. Diastolic (DA) and systolic (SA) areas of LV were measured in two-dimensional mode, using planimetry at the parasternal plane of the smaller axis.

LV systolic function was assessed by calculating the area variation fraction (AVF = (DA-SA)/DA) and the posterior wall shortening velocity (PWSV). Diastolic function was assessed by the E/A ratio, the E-wave deceleration time (EDT) and the Isovolumic Relaxation Time Adjusted for Heart Rate (IVRT/HR).^22,23^

### Histological analysis

After the echocardiographic study, the animals were euthanized and the hearts were removed and dissected. The right and left ventricles, including the interventricular septum, were separated. Cardiac tissue samples were fixed in a 10% formaldehyde solution for 48 hours, according to the previously described method.^24,25^

The histological sections were stained on slides with hematoxylin-eosin (HE) and Masson solution for assessment of the infarcted tissue, using a Leica DM LS microscope coupled to a video camera, which sends digital images to a computer with the Image Pro-Plus imaging analysis program (Media Cybernetics, Silver Spring, Maryland, USA).

The infarction size was determined in 5 to 6-mm sections from the apex, as the values in this region correspond to the mean values obtained from sections of the entire heart.^24,25^ To estimate the infarction size through histological analysis, the epicardial and endocardial circumferences of the infarcted and non-infarcted segments were determined. The infarction size is expressed as a percentage of the ventricular circumference measurements.

### Heart failure criteria

The diagnosis of heart failure was made by thrombus detection in the left atrium, pleural effusion, ascites, and right ventricular hypertrophy, characterized by the ratio of the right ventricle weight adjusted for body weight > 0.8 mg/g, as previously described.^4,26,27^

### Statistical analysis

The comparisons between the groups after 6 months were carried out using Student's *t* test when data showed normal distribution. When the data did not have normal distribution, comparisons between groups were performed using the Mann-Whitney U test. Data were expressed as mean ± standard deviation or median, with 25 and 75 percentiles. The predictive values were analyzed by logistic regression. In this analysis, the presence or absence of heart failure was used as the dependent variable. The cutoff values predictive of heart failure were determined using ROC curves. The significance level was set at 5%. Statistical analyses were performed using the SigmaPlot program for Windows, v.12.0 (Systat Software Inc., San Jose, CA, EUA).

## Results

Six months after surgery, 88 animals with infarction (I) and 43 control animals (C) were included in the study.

Echocardiographic variables are shown in Table 1. As expected, the infarction increased the left cavity diameters, LV mass and wall thickness. Additionally, the MI resulted in systolic and diastolic dysfunction, characterized by lower values of the AVF, PWSV, EDT, associated with higher values of E/A ratio and IVRT/HR.

**Table 1:** Echocardiographic study after six months of observation

**Variables**	**Control (n = 43)**	**AMI (n = 88)**	**p-value**
LAD (mm)	5.80 (5.55-6.10)	7.72 (6.70-8.59)	< 0.001
LVDD (mm)	8.39 (8.12-8.80)	10.96 (10.32-11.73)	< 0.001
LVSD (mm)	4.34 (4.08-4.66)	8.70 (7.61-9.75)	< 0.001
LVMI	1.94(1.74-2.11)	3.33 (2.79-4.09)	< 0.001
E/A	1.55 (1.40-1.69)	1.68 (1.32-4.85)	0.085
IVRT/HR	59.3 (5452-64.8)	70.4 (61.4-77.9)	< 0.001
EDT ( ms)	45 (41-55)	39 (33-48	< 0.001
AVF (%)	67 (64-71)	33 (35-36)	< 0.001
PWSV (mm/s)	37 (35-39)	25 (20-28)	< 0.001
PWDT	1.49 (1.43-1.59)	1.70 (1.59-1.87)	< 0.001

Data are expressed as median with 25 and 75 percentiles. AMI: animals with acute myocardial infarction; LAD: left atrium diameter; LVDD: left ventricular diastolic diameter; LVSD: left ventricular systolic diameter; LVMI: left ventricular mass index; E/A: E/A wave ratio; IVRT/HR: isovolumic relaxation time adjusted by heart rate; EDT: E-wave deceleration time; AVF: area variation fraction; PWSV: posterior wall shortening velocity; EDPP: posterior wall diastolic thickness.

Considering the infarcted animals, 54 animals (61%) developed heart failure. Animals with heart failure had larger infarctions (43.5 ± 7.5% vs. 40.0 ± 8.1%; p = 0.044), higher mass index and left cavity diameters, associated with worsening of functional variables, compared with the infarcted animals without heart failure (Table 2).

**Table 2:** Echocardiographic study of infarcted animals after 6 months of observation

**Variables**	**Without HF (n = 34)**	**With HF (n = 54)**	**p-value**
LAD (mm)	6.90 ± 1.29	8.16 ± 1.30	< 0.001
LVDD (mm)	10.6 ± 0.84	11.4 ± 1.05	< 0.001
LVSD (mm)	8.17 ± 1.13	9.02 ± 1.39	0.002
LVMI	3.14 (2.81-3.59)	3.64 (2.78-4.50)	0.018
E/A	1.43 (1.24-1.70)	3.91 (1.35-6.27)	0.002
IVRT/HR (ms)	73.9 ± 12.1	66.7 ± 12.0	0.008
EDT (ms)	43.1 ± 8.8	38.3 ± 10.2	0.036
AVF (%)	33.3 ± 8.6	29.6 ± 7.6	0.040
PWSV	25.9 (22.6-28.4)	24.2 (20.6-28.3)	0.344

Data are expressed as mean ± standard deviation (for normal distribution) or median with 25 and 75 percentiles (for non-normal distribution). HF: heart failure; LAD: left atrium diameter; LVDD: left ventricular diastolic diameter; LVSD: left ventricular systolic diameter; LVMI: left ventricular mass index; E/A: E/A wave ratio; IVRT/HR: isovolumic relaxation time adjusted by heart rate; EDT: E-wave deceleration time; AVF: area variation fraction; PWSV: posterior wall shortening velocity.

**Figure 1:**
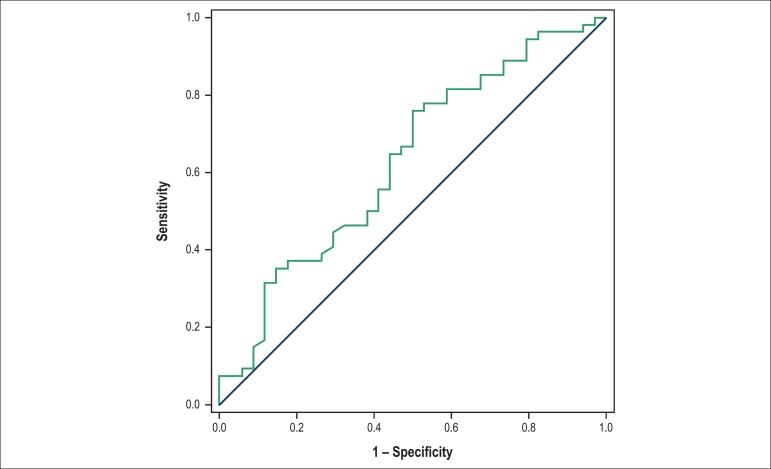
Cutoff value for the area variation fraction, as a heart failure predictor 6 months after infarction. Area under the curve: 0.6277; 95% confidence interval: 0.5066 to 0.7489; p-value: 0.044; cutoff < 31.18; sensitivity: 55.60%; specificity: 57.34%.

**Figure 2:**
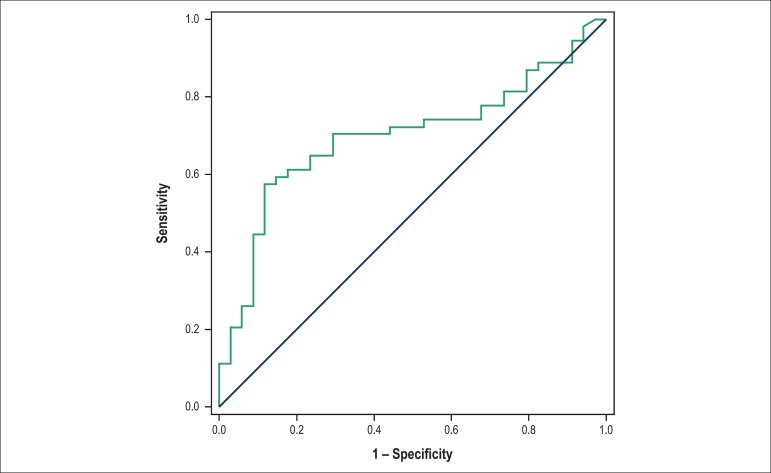
Cutoff value for the E/A ratio as a heart failure predictor 6 months after infarction. Area under the curve: 0.6985; 95% confidence interval: 0.5875 to 0.8095, p-value: 0.0017; cutoff > 3,077; sensitivity: 57.93%o; specificity: 62.56%o.

**Figure 3:**
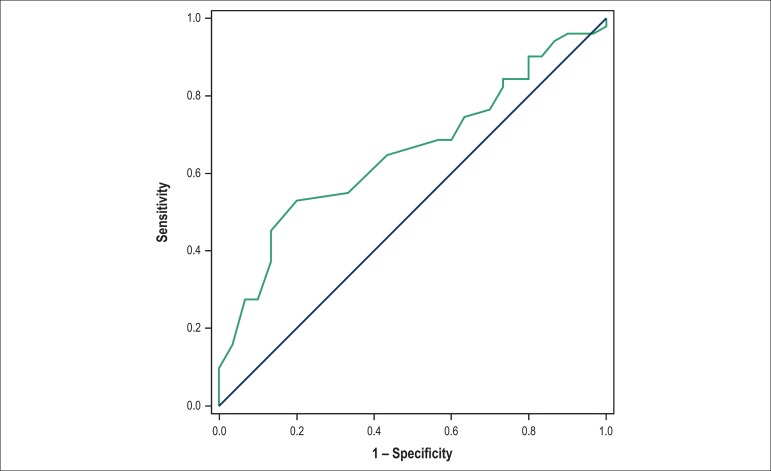
Cutoff value for the deceleration time of the E wave, as a heart failure predictor 6 months after infarction. Area under the curve: 0.6533; 95% confidence interval: 0.5341 to 0.7724; p-value: 0.0218; cutoff < 42.11; sensitivity: 59.77%; specificity: 51.85%.

**Figure 4:**
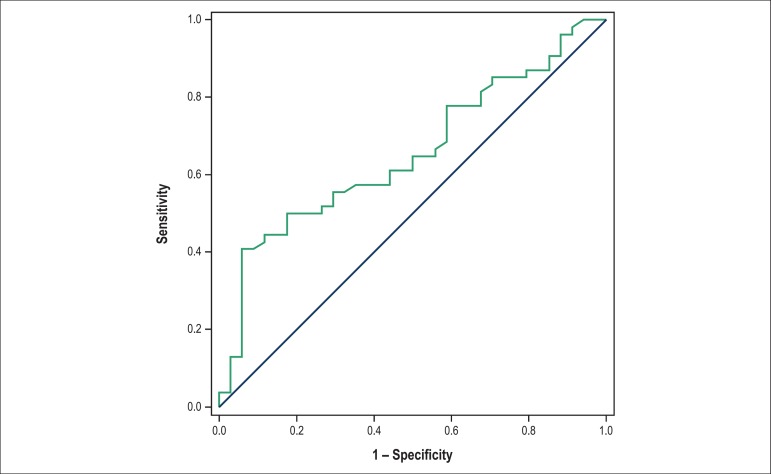
Cutoff value for the isovolumic relaxation time adjusted by heart rate as a heart failure predictor 6 months after infarction. Area under the curve: 0.6544; 95% confidence interval: 0.5398 to 0.7691; p-value: 0.01512; cutoff < 69.08; sensitivity: 55.10%; specificity: 60.49%.

Table 3 shows the results of the regression analyses. AVF, E/A ratio, EDT and IVRT/HR were the functional variables predictors of heart failure. However, we found that the predictive value was low for the functional variables, suggesting the importance of other variables. Additionally, the cutoff values of the functional variables associated with heart failure were: AVF: < 31.18% (Figure 1); E/A ratio > 3.077 (Figure 2); EDT: < 42.11 ms (Figure 3) and IVRT/HR: < 69.08 (Figure 4).

**Table 3:** Heart failure predictors 6 months after coronary occlusion

**Variables**	**OR**	**95%CI**	**p-value**
E/A	1.529	1.183-1.976	0.001
IVRT/HR	0.949	0.911-0.989	0.013
EDT (ms)	0.951	0.906-0.998	0.040
AVF (%)	0.944	0.892-0.999	0.045
PWSV (mm/s)	0.077	0.905-1.055	0.554

OR: odds ratio; 95%CI: 95% confidence interval; E/A: E wave/A wave ratio; IVRT/HR, isovolumic relaxation time adjusted by heart rate; DTE: deceleration time of E wave; AVF: area variation fraction; PWSV: posterior wall shortening velocity.

## Discussion

The aim of our study was to evaluate the functional variables associated with heart failure in an experimental MI model in rats. Our data suggested that AVF, E/A ratio, EDT and IVRT/HR are predictors of heart failure 6 months after infarction.

The first aspect to be considered is that in our study, most animals (61%) with infarction developed heart failure. In a previous study, we determined that infarctions affecting 40% of LV are required for the development of heart failure in this model.^26^ in accordance with this concept, in this study, the animals showed, on average, large infarcts. In this sense, we can infer that the coronary occlusion model in rats is appropriate for the study of the heart failure syndrome.

The second important aspect is related to the fact that, as expected, animals with heart failure had worsening of functional variables related to systolic function, when compared with animals without failure. However, in the regression analysis, the AVF, but not LVPW, was predictive of heart failure onset. It is believed that in models of regional LV akinesia, one-dimensional echocardiographic methods of functional assessment may be flawed. In this situation, it is recommended to use, for instance, the Simpson's method in humans. AVF is obtained through the analysis of the SA and DA, using the two-dimensional technique. However, the LVPW is obtained in single-dimensional mode. Therefore, our results emphasize that, in this model, similar to what occurs in humans, it is preferable to use systolic functional analysis with two-dimensional technique, such as AVF, for instance.

Another prominent aspect is related to the diastolic function. Unlike systolic function, the study of diastolic function through echocardiography in the rat model is not well standardized. Some of the main technical difficulties are the small size of the animal, with its implications on the transducer and heart rate of around 300 beats per minute. In our study, however, all assessed diastolic function variables were associated with heart failure onset. Thus, in this model, diastolic function assessed by E/A ratio, EDT and IVRT/HR were predictors of heart failure 6 months after coronary occlusion.

The most important aspect of our study is that heart failure prediction in the MI rat model has important implications. Although there is no consensus on the definition of cardiac dysfunction and heart failure, they are usually diagnosed by elevated end-diastolic pressure (PD_2_) of the LV (invasive hemodynamic method) and the presence of clinical signs assessed after death (RV hypertrophy, ascites, pleural effusion and left atrial thrombus), respectively. Therefore, our study suggests that echocardiography is a useful non-invasive tool for the prediction of this syndrome, as systolic function variables, as well the diastolic function parameters, little studied in this model, were associated with heart failure.

Previous studies have evaluated the association between echocardiographic parameters and heart failure. However, most studies have assessed the association between echocardiography and PD_2_, and not with heart failure clinical variables.^6-10^ Martinez et al.^5^ assessed the association between morphological and functional cardiac variables with the clinical manifestations of heart failure. However, the echocardiographic variables were studied through cluster analysis.^5^ Therefore, we believe that our study adds important information about the role of echocardiography as a predictor of long-term heart failure in this model.

Finally, it is already well-established that the major determinant of ventricular function, of the remodeling process and, consequently, of heart failure onset in this model, is the size of the infarction.^4,26,28,29^ In our study, however the difference in infarction size among animals with and without heart failure, although significant, was low (43 ± 7% vs. 40 ± 8%, respectively). Therefore, we conclude that other factors rather than the infarction size, are important determinants of heart failure onset in this model.

Among the possible candidates, we can include changes in ventricular cavity diameter, wall thickness alterations, changes in the normal LV configuration, from elliptical to the rounded shape, among others. Therefore, in some situations, changes in geometry alone could be responsible for ventricular global function impairment, by changing the load conditions to which the heart is submitted.

## Conclusion

In rats followed for 6 months after MI, the area variation fraction, the E/A ratio, E-wave deceleration time and the isovolumic relaxation time adjusted by heart rate were predictors of heart failure onset.
