# Erratum

**DOI:** 10.5935/abc.20160034

**Published:** 2016-03

## Issue of December 2014, vol. 103 (6), Supl. 2, pp. 1-126

In “I Diretriz Brasileira de Insuficiência Cardíaca e Transplante Cardíaco, no Feto, na Criança e em Adultos com Cardiopatia Congênita, da Sociedade Brasileira de Cardiologia”, consider Santos VG as the correct form for the name of the author Valter Garcia Santos.

## Issue of February de 2016, vol. 106 (2), pp. 105-112

In the original article “Association between Functional Variables and Heart Failure after Myocardial Infarction in Rats”, adjust the name of author Aline F. Lima to Aline R. Lima.

